# Dose selection for aztreonam-avibactam, including adjustments for renal impairment, for Phase IIa and Phase III evaluation

**DOI:** 10.1007/s00228-023-03609-x

**Published:** 2024-01-22

**Authors:** Shampa Das, Todd Riccobene, Timothy J. Carrothers, James G. Wright, Merran MacPherson, Andrew Cristinacce, Lynn McFadyen, Rujia Xie, Alison Luckey, Susan Raber

**Affiliations:** 1 AstraZeneca, Alderley Park, Macclesfield, UK; 2 Present Address: Department of Molecular and Clinical Pharmacology, University of Liverpool, Liverpool, UK; 3 AbbVie, Madison, NJ USA; 4 Present Address: Intra-Cellular Therapies, Inc, New York, NY USA; 5 Wright Dose Ltd, Altrincham, Cheshire UK; 6 Present Address: UCB, Braine-l’Alleude, Wallonia, Belgium; 7 Pfizer, Sandwich, Kent UK; 8 Present Address: Canterbury, Kent, UK; 9 Pfizer, Singapore; 10 Pfizer, New York, NY USA; 11 Present Address: GARDP (Global Antibiotics Research & Development Partnership), Geneva, Switzerland; 12 Global Product Development, Pfizer Inc, 10555 Science Center Dr, San Diego, CA 92121 USA

**Keywords:** Infection, Pharmacodynamics, Anti-bacterials, Antibiotics, Dosing

## Abstract

**Purpose:**

A series of iterative population pharmacokinetic (PK) modeling and probability of target attainment (PTA) analyses based on emerging data supported dose selection for aztreonam-avibactam, an investigational combination antibiotic for serious Gram-negative bacterial infections.

**Methods:**

Two iterations of PK models built from avibactam data in infected patients and aztreonam data in healthy subjects with “patient-like” assumptions were used in joint PTA analyses (primary target: aztreonam 60% *f*T > 8 mg/L, avibactam 50% *f*T > 2.5 mg/L) exploring patient variability, infusion durations, and adjustments for moderate (estimated creatinine clearance [CrCL] > 30 to ≤ 50 mL/min) and severe renal impairment (> 15 to ≤ 30 mL/min). Achievement of > 90% joint PTA and the impact of differential renal clearance were considerations in dose selection.

**Results:**

Iteration 1 simulations for Phase I/IIa dose selection/modification demonstrated that 3-h and continuous infusions provide comparable PTA; avibactam dose drives joint PTA within clinically relevant exposure targets; and loading doses support more rapid joint target attainment. An aztreonam/avibactam 500/137 mg 30-min loading dose and 1500/410 mg 3-h maintenance infusions q6h were selected for further evaluation. Iteration 2 simulations using expanded PK models supported an alteration to the regimen (500/167 mg loading; 1500/500 mg q6h maintenance 3-h infusions for CrCL > 50 mL/min) and selection of doses for renal impairment for Phase IIa/III clinical studies.

**Conclusion:**

A loading dose plus 3-h maintenance infusions of aztreonam-avibactam in a 3:1 fixed ratio q6h optimizes joint PTA. These analyses supported dose selection for the aztreonam-avibactam Phase III clinical program. *Clinical trial registration*: NCT01689207; NCT02655419; NCT03329092; NCT03580044.

**Supplementary Information:**

The online version contains supplementary material available at 10.1007/s00228-023-03609-x.

## Introduction

Aztreonam-avibactam is an investigational fixed-dose combination antibiotic for serious infections caused by Gram-negative bacteria for which there are limited or no treatment options. Carbapenem-resistant *Enterobacterales* (CRE), including those that produce Ambler class B metallo-β-lactamases (MBLs), are an emerging threat for which treatment options are severely limited [1–4]. Aztreonam is an established monobactam (β-lactam), which retains activity in the presence of Ambler class B MBLs but is vulnerable to other β-lactamases which can be inhibited by avibactam, a first-in-class β-lactamase inhibitor. Avibactam has also been successfully partnered with ceftazidime (in a 4:1 fixed-dose ratio) for the treatment of infections caused by non-MBL CRE and other Gram-negative bacteria [5–8].

Initial clinical evaluations of the aztreonam-avibactam combination consisted of a Phase I dose-finding, safety, and pharmacokinetic (PK) trial in healthy subjects (NCT01689207) and a Phase IIa trial (REJUVENATE [NCT02655419]) in patients with complicated intra-abdominal infection (cIAI) [9, 10]. Two further confirmatory PK trials (NCT04486625 and NCT04973826) and two Phase III trials in patients with cIAI, complicated urinary tract infections (cUTI), hospital-acquired pneumonia and ventilator-associated pneumonia, and bloodstream infections caused by Gram-negative pathogens, including MBL-producing pathogens (REVISIT [NCT03329092] and ASSEMBLE [NCT03580044]), have recently been completed [11].

Clinical development of aztreonam-avibactam has been streamlined by leveraging preclinical and in vitro microbiological data [12–15], prior pharmacokinetic (PK) knowledge of aztreonam monotherapy [16–19], and avibactam population PK models built during the ceftazidime-avibactam clinical development program [5, 20–24]. Dose regimen selection for the aztreonam-avibactam clinical program was guided by approved dosing recommendations for aztreonam monotherapy, in vitro minimum inhibitory concentration (MIC) distributions for aztreonam and avibactam in combination against target pathogens, and simulations of joint probability of PK-pharmacodynamic (PD) target attainment (PTA) evaluating a range of dosing scenarios, balanced with exposures selected to limit potential adverse effects.

For β-lactam/β-lactamase inhibitor combinations, simultaneous achievement of both drugs’ respective plasma targets (referred to as joint target attainment) is required for antibacterial efficacy. For time-dependent drugs such as β-lactams, the PK/PD driver for efficacy is the proportion of the dosing interval that free plasma concentrations exceed the MIC (i.e. %*f*T > MIC). For avibactam, the time that free plasma concentrations exceed a critical threshold concentration (%*f*T > C_T_) is the PK/PD driver associated with efficacy in combinations with aztreonam or the cephalosporins ceftazidime and ceftaroline [13]. However, the threshold concentration required to protect the partner β-lactam varies depending on the combination [25]. Notably, the target for avibactam with aztreonam (50% *f*T > 2.5 mg/L) is 2.5-fold higher than for avibactam with ceftazidime (50% *f*T > 1 mg/L) [12, 13, 25]; this has fundamental implications for dosing of the respective combinations. Aztreonam is eliminated by both renal and extra-renal routes (approximately 70% and 30%, respectively) [26, 27], and dose adjustments are made for patients with severe renal impairment, defined as estimated creatinine clearance (CrCL) < 30 mL/min [28, 29]. In contrast, avibactam is almost entirely eliminated by renal clearance (97%), and doses of ceftazidime-avibactam are therefore modified for patients with estimated CrCL < 50 mL/min [30, 31]. Since the extent of renal clearance of avibactam is greater than that of aztreonam, understanding the relationships between renal function and exposures of both drugs has been an important aspect of the development program.

This paper describes the process of iterative population PK modeling and PTA analyses which, as additional PK and clinical data became available, supported dose selection and modification/optimization for the aztreonam-avibactam Phase II and Phase III clinical program. This included evaluation of loading doses and extended infusions, and dose adjustments for patients with moderate and severe renal impairment, accounting for the differential renal clearance of aztreonam and avibactam.

## Methods

### Overview and chronology of population PK modeling analyses

The aztreonam-avibactam clinical trial program has evaluated a range of intravenous dosing regimens (Table 1); an iterative series of population PK modeling and simulation analyses based on emerging data (Table 2) supported selection of the doses for the final cohorts of a Phase I “first-in-human” trial and for the Phase IIa and Phase III trials of aztreonam-avibactam. The Phase I trial was a three-part, placebo-controlled safety and PK evaluation of the combination of aztreonam with avibactam in healthy subjects [9]. In Part A, single doses of aztreonam 2000 mg with and without avibactam 600 mg were administered by 1-h infusions. These evaluations used the approved dose of aztreonam alone (2 g every 6–8 h) for severe infections in patients with normal renal function [28, 29] and avibactam doses supported by preclinical experiments to determine PK-PD targets, which used a human-simulated dose of 2000 mg aztreonam combined with 375 or 600 mg of avibactam [12, 13]. In Part B Cohorts 1 and 2, aztreonam-avibactam doses of 2000/375 mg and 2000/600 mg 1-h infusions every 6 h (q6h) were evaluated. Potentially clinically significant plasma aspartate transaminase (AST) and alanine transaminase (ALT) elevations prompted the early termination of enrolment in Part B Cohort 2. In Part B Cohort 3, the aztreonam dose was reduced to 1500 mg with avibactam 600 mg administered as 2-h infusions q6h. Although stopping criteria were not met, the sponsor temporarily stopped enrolment again due to transaminase elevations.

**Table 1:** Overview of dose regimens evaluated in the aztreonam-avibactam clinical trial program

**Trial**	**Population and design**	**Dosing regimens (infusion duration)**		
**Phase I **[9]	Healthy subjects^a^	**Normal renal function/mild impairment (CrCL > 50 mL/min)**		
**Dose-finding (NCT01689207)**	Randomized, placebo-controlled	**Part A** Single doses: 2000 mg aztreonam and/or 600 mg avibactam (1-h)	**Part B** Cohort 1: 2000/375 mg (1-h) q6h Cohort 2: 2000/600 mg (1-h) q6h Cohort 3: 1500/600 mg (2-h) q6h Cohort 4: 1500/450 mg (3-h) q6h Cohort 5: 1500/410 mg (3-h) q6h	**Part C** LD: 500/137 mg (30-min) ELD: 1500/410 mg (2.5-h) MD: 1500/410 mg (3-h) q6h
**Phase IIa **[10]	Patients with cIAI^**a**^	**Normal renal function/mild impairment (CrCL > 50 mL/min)**		**Moderate renal impairment (CrCL > 30 to 50 mL/min)**
**REJUVENATE (NCT02655419)**	Non-randomized, open-label^b,^ ^c^	**Cohort 1** LD: 500/137 mg (30-min) ELD: 1500/410 mg (3-h) MD: 1500/410 mg (3-h) q6h	**Cohorts 2 and 3**^**c**^ LD: 500/167 mg (30-min) ELD: 1500/500 mg (3-h) MD: 1500/500 mg (3-h) q6h	**Cohorts 2 and 3**^**c**^ LD: 500/167 mg (30-min) ELD: 1500/500 mg (3-h) MD: 750/250 mg (3-h) q6h
**Phase III**	Patients with cIAI, cUTI, BSI or HAP/VAP^a^	**Normal renal function/mild impairment (CrCL > 50 mL/min)**	**Moderate renal impairment (CrCL > 30 to 50 mL/min)**	**Severe renal impairment (CrCL > 15 to 30 mL/min)**
**REVISIT (NCT03329092)** **ASSEMBLE (NCT03580044)**	Randomized, active comparator^b^	LD: 500/167 mg (30-min) ELD: 1500/500 mg (3-h) MD: 1500/500 mg (3-h) q6h	LD: 500/167 mg (30-min) ELD: 1500/500 mg (3-h) MD: 750/250 mg (3-h) q6h	LD: 675/225 mg (30-min) ELD: 675/225 mg (3-h) MD: 675/225 mg (3-h) q8h

*BSI* bloodstream infection, *cIAI* complicated intra-abdominal infection, *cUTI* complicated urinary tract infection, *CrCL* creatinine clearance, *ELD* extended loading dose, *HAP/VAP* hospital-acquired pneumonia/ventilator-associated pneumonia, *LD* loading dose, *MD* maintenance dose, *q6h* every 6 h, *q8h* every 8 h

^a^Phase I Part A, Part B, and Part C Cohort 1 enrolled subjects ≥ 18 to 45 years old. Phase 1 Part C Cohort 2 enrolled subjects ≥ 65 years old; age inclusion criteria for Phase IIa and Phase III include patients ≥ 18 to ≤ 90 years old, and ≥ 18 years old, respectively. Subjects in Part A received single doses of aztreonam 2000 mg, avibactam 600 mg and aztreonam-avibactam 2000–600 mg or matching placebo IV infusions by randomized crossover, each separated by a ≥ 3-day washout period. Subjects in Part B received 11 days of aztreonam-avibactam or matching placebo IV infusions q6h on days 2–10; single doses were administered on Days 1 and 11. Subjects in Part C received 10 days of aztreonam-avibactam or matching placebo, with single doses on Day 10

^b^Recommended treatment duration in the Phase IIa and Phase III trials: 5–14 days for patients with cIAI, cUTI, or BSI; and 7–14 days for patients with HAP/VAP. Patients with cIAI treated with aztreonam-avibactam received concomitant metronidazole 500 mg IV q8h for anaerobic pathogen coverage

^c^Selection of the dosing regimen used in Cohorts 2 and 3 was subject to recommendations from the Scientific Advisory Committee, following planned reviews of safety and PK data from Cohorts 1 and 2. Patients in Cohort 2 with estimated CrCL > 50 mL/min subsequently received the aztreonam-avibactam 1500/500 mg q6h maintenance dose regimen. Cohort 3 used the same 1500/500 mg q6h maintenance dose regimen for patients with CrCL > 50 mL/min, with the addition of an adjusted regimen for patients with estimated CrCL > 30 to 50 mL/min

**Table 2:** Population PK model iterations, data sources, modeling objectives, and assumptions

**Population PK model**	**Numbers of subjects (number of samples)**	**Modeling objectives and simulations**	**Key assumptions**
**Aztreonam Iteration 1a** Aztreonam-avibactam Phase I dose-finding trial (interim data) [18]	27 (819)	Dose selection for Phase I Part B (Cohorts 4 and 5) and Part C, and Phase IIa Cohort 1 Simulations for extended 3-h infusion vs CI (normal renal function) Simulations for loading dose (CI; normal renal function)	Various aztreonam PK scenarios evaluated in Iteration 1a simulations, including patient factors for patients with CF and cIAI (see Supplementary Table 1) Patient factors for Phase II patients with cIAI and variability from avibactam models in the ceftazidime-avibactam program (Case 4) used for Iteration 1b simulations
**Aztreonam Iteration 1b** Aztreonam-avibactam Phase I dose-finding trial (interim data) [18] Published aztreonam renal impairment studies [32, 33]	69 (1236)		
**Avibactam Iteration 1** Ceftazidime-avibactam program: 5 Phase I trials, 1 Phase II trial in cIAI Aztreonam-avibactam Phase I dose-finding trial (interim data) [18]	315 (5442)		
**Aztreonam Iteration 2** Aztreonam-avibactam Phase I dose-finding trial (final data) [18] Published aztreonam renal impairment studies [32, 33]	107 (2626)	Dose modification for Phase IIa Cohorts 2 and 3, dose selection for Phase III Simulations for normal renal function and mild, moderate and severe renal impairment in patients with APACHE II score ≤ 10	Scaling of aztreonam V_c_ larger in patients than healthy volunteers i.e., + 27.5% as per avibactam Patient factors and variability from avibactam models in ceftazidime-avibactam program (Phase I, Phase II cIAI, Phase III cIAI and cUTI) Simulations used ceftazidime-avibactam Phase III age, weight, and CrCL covariate distributions Nonlinear plasma protein binding for aztreonam
**Avibactam Iteration 2** Ceftazidime-avibactam program: 11 Phase I trials, 2 Phase II trials, 4 Phase III trials [21, 22]	1836 (12,499)		

*APACHE II* Acute Physiology and Chronic Health Evaluation II, *CI* continuous infusions, *CF* cystic fibrosis, *cIAI* complicated intra-abdominal infection, *cUTI* complicated urinary tract infection, *CrCL* creatinine clearance

Following these clinical observations, early population PK models (designated Iteration 1) were developed. For aztreonam, the Iteration 1 model utilized interim PK data from the Phase I study supplemented with published data on aztreonam alone [32, 33]. For avibactam, the Iteration 1 model included data from the ceftazidime-avibactam program [21, 22] in addition to the Phase I PK data. Exposure and joint PTA simulations were performed from the Iteration 1 model to support selection of reduced doses for Phase I Part B Cohorts 4 and 5, and Part C. Since all of the aztreonam PK data included in the model was derived from only healthy subjects, various aztreonam “patient-like” PK assumptions were explored in these simulations (Supplementary Table 1). The final regimen evaluated in Phase I Part C comprised an aztreonam-avibactam loading dose of 500/137 mg (30-min infusion) immediately followed by an extended loading dose with subsequent maintenance doses of 1500/410 mg (3-h infusions) q6h; this regimen was also evaluated (with a minor modification) in Phase IIa Cohort 1 [10].

During enrolment in Phase IIa Cohort 1, the aztreonam and avibactam population PK models were updated (Iteration 2), which included final data from the above Phase 1 trial. For aztreonam, the Iteration 1 model contained additional published PK data in healthy subjects and subjects with renal impairment, but did not include data from patients with cIAI or cUTI. Thus for Iteration 2, variances and covariates for aztreonam were imputed based on the established avibactam models developed during the ceftazidime-avibactam clinical program, which incorporated data from Phase II and Phase III patients. While these variances and covariates are expected to differ between the two drugs, this approach was considered more realistic than relying solely on aztreonam PK variability in healthy volunteers. As both drugs share a primary elimination pathway, it was reasonable to include for aztreonam assumptions based on variability in avibactam PK, which has been well explored in the target patient population. From the Iteration 2 model, additional simulations were conducted to support dose regimen selection for Phase IIa (Cohorts 2 and 3) and for subsequent Phase III evaluation. These simulations also included adjustments for patients with moderate and severe renal impairment.

### Data sources

Data sources, including numbers of PK samples, and key objectives and assumptions used for the two aztreonam and avibactam population PK model iterations are shown in Table 2. The aztreonam population PK models used subject PK data from the Phase I aztreonam-avibactam trial [9] and two published studies of aztreonam in healthy subjects and subjects with renal impairment [32, 33]. Both avibactam model iterations used PK data obtained during the ceftazidime-avibactam clinical program, including healthy and renally impaired subjects and patients with cIAI (and cUTI in Iteration 2) [20–22]. The Phase I trials (of avibactam ± aztreonam or avibactam ± ceftazidime) included intensive blood sampling for PK analysis (up to 17 samples per subject per day), and the Phase II and III trials (of ceftazidime-avibactam) included sparse PK sampling protocols (up to four samples per patient per day) for blood collection mainly on Day 3. In the aztreonam renal impairment studies, 10–12 PK samples per subject were obtained over 24–48 h [32, 33]. Baseline and demographic characteristics of subjects in each model are summarized in Table 3.

**Table 3:** Baseline and demographic characteristics of subjects in each population PK model iteration

	**Aztreonam**			**Avibactam**	
	**Iteration 1a**	**Iteration 1b**	**Iteration 2**	**Iteration 1**	**Iteration 2**
**Numbers of subjects**	27	69	107	315	1836
**Age, years**	30 (18–45)	33 (18–64)	33 (18–74)	33 (18–80)	48 (18–89)
**Sex, *n* (%)**					
M﻿ale	27 (100)	69 (100)	105 (98.2)	245 (78.8)	1025 (55.8)
Female	0	0	2 (1.8)	70 (22.2)	811 (42.2)
**Body weight, kg**	74 (63–82)	76 (48–97)	77 (48–102)	72 (41–127)	71 (28–171)
**Population, *n* (%)**					
Phase I/published literature	27 (100)	65 (100)	107 (100)	257 (81.6)	345 (18.8)
cIAI–Phase II	–	–	–	58 (18.4)	58 (3.2)
cIAI–Phase III	–	–	–	–	702 (38.2)
cUTI–Phase II	–	–	–	–	84 (4.6)
cUTI–Phase III	–	–	–	–	647 (35.2)
**Baseline CrCL, mL/min**	125 (89–213)	102 (5–213)	116 (5–232)	111 (14–219)	99 (8–384)
**Renal function group (CrCL), n (%)**					
Normal (> 80 mL/min)	27 (100)	39 (56.5)	76 (71.0)	251 (79.7)	1232 (67.1)
Mild (> 50 to 80 mL/min)	–	4 (5.8)	5 (4.7)	43 (13.7)	421 (22.9)
Moderate (> 30 to 50 mL/min)	–	5 (7.3)	5 (4.7)	13 (4.1)	147 (8.0)
Severe (> 15 to 30 mL/min)	–	9 (13.0)	9 (8.4)	7 (2.2)	31 (1.7)
Very severe (≤ 15 mL/min)	–	12 (17.4)	12 (11.2)	1 (0.3)	5 (0.3)

Continuous variables are shown as median (range)

*cIAI* complicated intra-abdominal infection, *cUTI* complicated urinary tract infection, *CrCL* creatinine clearance

### Bioanalytical methods

For subject PK data from the aztreonam-avibactam and ceftazidime-avibactam clinical programs, plasma samples were assayed by Covance Bioanalytical Laboratory, Harrogate, UK, using validated liquid chromatographic-tandem mass spectrometric (LC–MS/MS) methods. The lower limits of quantification were 0.1 mg/L for aztreonam and 0.01 mg/L for avibactam. The plasma concentration data for aztreonam taken from literature used a microbiological agar diffusion method with *Escherichia coli* (test organism) with a lower limit of quantification of 0.04 mg/L [32, 33].

### Modeling software

Population PK modeling used the FOCE-I method in NONMEM v7.2.0 or higher (Icon Development Solutions, Ellicott City, MD, USA). Simulations were performed with NONMEM and/or R v3.3.0 or higher (https://www.r-project.org/).

### Population PK model development

#### Aztreonam

A two-compartment model (Supplementary Table 2) was fitted to interim data from 27 healthy volunteers from the Phase I aztreonam-avibactam trial (Iteration 1a) and subsequently a total of 69 subjects from both the Phase I trial and two published studies (Iteration 1b), to include the effect of renal impairment. Renal impairment was modeled with a power model for CrCL on clearance (CL).

For model Iteration 2, the two-compartment disposition model was fitted to data from 107 healthy subjects and subjects with renal impairment; all parameters used standard allometry (exponents of 1 for volumes and 0.75 for CL and inter-compartmental clearance [Q] respectively; Supplementary Table 3). Covariate effects of CrCL on CL (two-part linear functions with steeper slope for CrCL < 80 mL/min) and age (> 50 or > 65 years) on apparent volume of the central compartment (*V*_*c*_) and apparent volume of the peripheral compartment (*V*_*p*_), and nonlinearity of aztreonam protein binding (*B*_max_, B_50_) were included.

#### Avibactam

For model Iteration 1, a two-compartment model was fitted to data from 315 healthy subjects, subjects with renal impairment and patients with cIAI, with covariate effects for CrCL on CL and for cIAI patients on both CL (45% increase) and *V*_*c*_ (166% increase) compared with healthy subjects (Supplementary Table 4).

For model Iteration 2, an updated two-compartment model was fitted to data from 1836 healthy subjects, subjects with renal impairment, and patients with cIAI or cUTI from the ceftazidime-avibactam development program (Supplementary Table 5). The model no longer included an adjustment for CL as seen for Phase II cIAI patients; Phase III cIAI patient effect on *V*_*c*_ remained (27.5% increase) but was smaller than that for Phase II patients with cIAI.

### Exposure and PTA analyses

The two sets of population PK model iterations were used to simulate exposures and joint PTA for various aztreonam-avibactam dose regimens; simulations based on Iteration 1 explored the impact of different scenarios for patient covariate effects as described below, and those based on Iteration 2 included evaluations of the impact of renal function (normal, mild, moderate, and severe renal impairment). Exposures were calculated from total plasma concentrations, and PTA simulations used free (unbound) fractions for aztreonam and avibactam of 58% [18] and 92%, respectively [30].

#### PK-PD targets

In vitro global surveillance studies have reported that > 99% of clinically relevant *Enterobacterales* isolates, including MBL producers, are inhibited at aztreonam-avibactam MICs ≤ 8 mg/L (with avibactam fixed at 4 mg/L) [34–38]; ≤ 8 mg/L is also the tentative susceptible MIC breakpoint against target Gram-negative bacteria for the aztreonam-avibactam combination. Plasma PK-PD targets for the aztreonam and avibactam combination against MBL- and extended-spectrum β-lactamase (ESBL)-producing clinical *Enterobacterales* isolates have been derived from an in vitro hollow fiber infection model (HFIM) [13] with validation of the avibactam target in an in vivo mouse thigh model [12]; a 60% *f*T > MIC value for aztreonam was the exposure measure most closely associated with efficacy [25]. Experiments using fixed doses of aztreonam in these models identified a C_T_ for avibactam of 2.5 mg/L; a target of 50% *f*T was within the range of efficacy estimates for the HFIM [13] and was considered conservative compared with the murine model (maximal effect of avibactam [combined with aztreonam, with aztreonam exposure fixed at > 50% *f*T > MIC] against MBL- and ESBL-producing *E. coli* and *Klebsiella pneumoniae* isolates where efficacy was achieved at 35–40% *f*T = 2.0–2.5 mg/L) [13]. Based on these studies, a joint PK-PD target (defined as attainment of 60% *f*T > MIC for aztreonam, and 50% *f*T > 2.5 mg/L for avibactam, achieved simultaneously) was evaluated in the current PTA analyses, with MIC = 8 mg/L considered as the primary target.

#### Iteration 1 simulations for Phase I dose modification and Phase IIa dose selection

Simulations using Iteration 1 population PK aztreonam and avibactam models (Supplementary Tables 2 and 4, respectively) evaluated various aztreonam-avibactam dose regimens (1000 subjects per simulation), including extended 3-h infusions and 6-h (continuous) infusions q6h, and loading doses in subjects with normal renal function. The aim of these simulations was to select a dose regimen that provided adequate exposures and joint PTA ≥ 90% at MIC = 8 mg/L while attempting to reduce the frequency of transaminase elevations observed in earlier cohorts of the Phase I trial [9]. Since the only available aztreonam PK data were from healthy subjects, five scenarios (designated Cases 1–5; Supplementary Table 1) encompassing additional assumptions on the influences on patient PK on key PK parameters were explored. This included using the CL value for patients with cystic fibrosis (Cases 2 and 3). Patients with cystic fibrosis have been reported to have increased aztreonam CL values, as well as increased variability, relative to healthy individuals [18]. In Cases 4 and 5, alterations in CL and *V*_*c*_ compared to healthy volunteers and associated variances observed for avibactam and ceftazidime in cIAI patients were applied to aztreonam [20].

#### Iteration 2 simulations for Phase IIa dose optimization and Phase III dose selection

The Iteration 2 population PK aztreonam and avibactam models (Supplementary Tables 3 and 5, respectively) were used to simulate exposure and joint PTA for various aztreonam-avibactam dose regimens in patients with cIAI with normal renal function and mild, moderate, or severe renal impairment (estimated CrCL > 80 mL/min, > 50 to ≤ 80 mL/min, > 30 to ≤ 50 mL/min, and > 15 to ≤ 30 mL/min, respectively). The aim was to optimize doses for Phase IIa and Phase III patients with normal renal function, and to select regimens for patients with moderate and severe renal impairment. In the absence of aztreonam patient PK data, the aztreonam simulation model was adapted from that shown in Supplementary Table 3 to include an increase of *V*_*c*_ for patients with cIAI of 27.5% (based on avibactam data from Phase III patients with cIAI), with no change to CL. Aztreonam parameter covariance was assumed to be the same as for the Iteration 2 avibactam model, leading to a greater dispersion across patients.

The avibactam simulation model [21] used Phase III cIAI parameters for *V*_*c*_ with the major covariate of CrCL on CL. Covariates of age, weight, and CrCL from Phase III patients in the ceftazidime-avibactam program [22] were used to create multivariate covariate distributions. Exposure and joint PTA simulations were conducted for 5000 simulated patients for each renal function group and dose regimen by resampling these distributions. For simulations of patients with moderate and severe renal impairment, CrCL values were assumed to follow a uniform distribution within the designated range for each category.

Dose regimen selection for patients with normal renal function or mild renal impairment was based on achieving ≥ 90% joint PTA in simulated patients with cIAI based on the above PK-PD targets and consideration of the safety profile. A variety of dose regimens for potential clinical evaluation in patients with moderate and severe renal impairment, maintaining a fixed aztreonam to avibactam dose ratio across renal function groups, were evaluated using the following criteria: (1) matching daily aztreonam area under the plasma concentration curve (AUC_(0–24)_) to the normal renal function group (without avibactam exposure exceeding that in the mild renal impairment group); and (2) joint PTA ≥ 90% at MIC = 8 mg/L, taking into account the differential effects of renal impairment on aztreonam and avibactam (matching AUC_(0–24)_ for aztreonam to normal renal function would implicitly result in exceeding the corresponding avibactam exposures in normal renal function; this was deemed acceptable given the known safety profile of avibactam).

## Results

### Exposure and PTA analyses for Phase I dose modification and Phase IIa dose selection

**Fig. 1:**
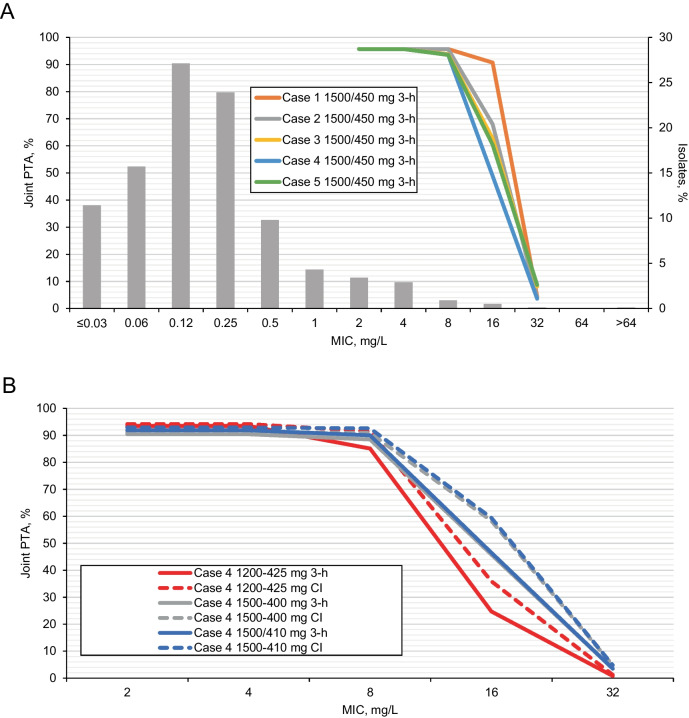

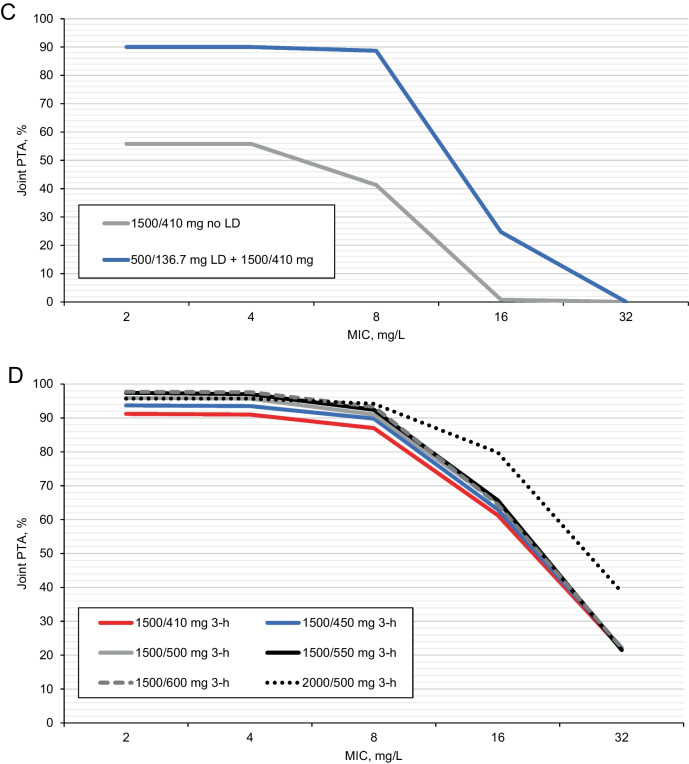
Joint PTA by MIC at steady-state for selected aztreonam-avibactam dose regimens in simulated subjects with normal renal function^a^. **A** Aztreonam-avibactam 1500/450 mg maintenance doses (3-h infusions q6h): Impact of aztreonam PK scenarios (aztreonam PK model Iteration 1a / avibactam PK model Iteration 1), overlaid with aztreonam-avibactam MIC distribution against MBL-producing *Enterobacterales* collected during 2016–2020 as part of the ATLAS Global Surveillance Program^b^. **B** Potential aztreonam-avibactam maintenance doses: 3-h infusions q6h vs continuous infusions (aztreonam PK model Iteration 1b / avibactam PK model Iteration 1)^c^. **C** Joint PTA during the first 6-h dosing interval on day 1 for aztreonam-avibactam 1500/410 mg q6h (continuous infusions) with and without loading dose 500/137 mg (aztreonam PK model Iteration 1b / avibactam PK model Iteration 1)^c^. **D** Potential aztreonam-avibactam maintenance doses (3-h infusions q6h): joint PTA with varying aztreonam and avibactam doses (aztreonam PK model Iteration 2 / avibactam PK model Iteration 2)^d^. *CI* continuous infusions, *cIAI* complicated intra-abdominal infection, *CL* clearance, *IIV* inter-individual variability, *MIC* minimum inhibitory concentration, *PTA*, probability of target attainment, *q6h*, every 6 h, *V*_*c*_ apparent volume of the central compartment. ^a^Probability of attaining the joint PK-PD target, defined as free plasma concentrations of aztreonam above the specified MIC for 60% of each dosing interval (60% *f*T > MIC mg/L) and free concentrations of avibactam above 2.5 mg/L for 50% of each dosing interval (50% *f*T > 2.5 mg/L). Both targets needed to be achieved simultaneously to meet the joint target. Joint PTA at steady-state is reported unless stated otherwise. ^b^Aztreonam PK scenarios (Supplementary Table 1): Case 1, healthy subjects’ CL and IIV (5.5 L/h and 8.8%, respectively; Supplementary Table 1); Case 2, CL for patients with cystic fibrosis (6.1 L/h) [18] and IIV of 17.6% (i.e., doubled from healthy subjects in Case 1); Case 3, CL for patients with cystic fibrosis (6.1 L/h) [18] and IIV of 27.9% (equal to that of avibactam; Supplementary Table 3); Case 4, 45% increase in CL and 166% increase in *V*_*c*_, as observed in Phase II patients with cIAI for avibactam; IIVs as for avibactam (27.9% and 32.1%; Supplementary Table 3); and Case 5, 23% increase in CL and 108% increase in *V*_*c*_, as observed in patients with cIAI for ceftazidime; respective IIVs of 37.4% and 49.0% from the early ceftazidime PK model [20]. ^c^Aztreonam PK scenario Case 4 (Supplementary Table 1). ^d^Simulations included a loading dose of one-third of the maintenance dose given by 30-min infusion. MIC distributions reproduced with permission [37]

Simulations based on the Iteration 1 population PK models were used to select aztreonam-avibactam maintenance dose regimens for Phase I Part B Cohorts 4 and 5 and Part C [9], with the aim of achieving adequate PTA with reduced doses and increased infusion durations. For PK assumptions based on healthy subjects (Case 1; see Supplementary Table 1), aztreonam-avibactam 1500/450 mg maintenance 3-h infusions q6h achieved joint PTA > 90% at MICs ≤ 16 mg/L; with ‘patient-like’ aztreonam PK assumptions (Cases 2–5), joint PTA was > 90% for MICs ≤ 8 mg/L (Fig. 1A). Of note, achievement of joint PTA ≥ 90% for MICs < 8 mg/L in these scenarios was driven primarily by the avibactam dose, with avibactam maintenance doses > 400 mg q6h needed for Cases 2–5 (data not shown). Based on these analyses, an aztreonam-avibactam maintenance dose regimen of 1500/450 mg 3-h infusions q6h was selected for Part B Cohort 4. Case 4 (cIAI patient-like) PK assumptions for aztreonam resulted in the most variability (and therefore the lowest joint PTA), and Case 4 was therefore the main focus in subsequent simulations.

Following completion of Part B Cohort 4, simulations explored reductions in the aztreonam and/or avibactam component of the combined aztreonam-avibactam dose, and comparing 3-h infusions with continuous infusions, to select a dose regimen for Part B Cohort 5. In these analyses, achievement of joint PTA at MIC = 8 mg/L was driven primarily by the avibactam dose, with no meaningful differences in joint PTA for 3-h infusions q6h compared with continuous infusions at steady-state (Fig. 1B). It was apparent from these simulations that addition of a 30-min infusion loading dose of aztreonam-avibactam 500/137 mg was required to improve joint PTA during the first 6 h for continuous infusion (Fig. 1C). An aztreonam-avibactam maintenance dose regimen of 1500/410 mg 3-h infusions q6h was selected for Part B Cohort 5, with loading doses of 500/137 mg 30-min infusion, immediately followed by a 1500/410 mg 2.5-h infusion (extended loading dose), followed by 1500/410 mg 3-h infusions q6h (maintenance dose). This regimen was further evaluated in Phase I Part C. This final Phase I dose regimen was adapted for evaluation in cIAI patients with estimated CrCL > 50 mL/min in Phase IIa Cohort 1 to give the extended loading dose by 3-h infusion, a minor change for clinical acceptability with no meaningful impact on exposure or joint PTA.

### Exposure and PTA analyses for Phase IIa dose optimization and Phase III dose selection

During enrolment in Cohort 1 of the Phase IIa study of aztreonam-avibactam in patients with cIAI [10], additional simulations were performed using Iteration 2 population PK models to select doses for evaluation in Phase IIa Cohorts 2 and 3 and for the Phase III trials, including doses for patients with moderate and severe renal impairment. Predicted steady-state exposures and joint PTA at MIC = 8 mg/L for the selected dose regimens are shown in Table 4.

**Table 4:** Predicted steady-state median exposures and joint PTA at MIC = 8 mg/L for aztreonam-avibactam dose regimens evaluated in the Phase IIa and Phase III clinical trials (PK model Iteration 2)

**Trial**	**Renal function group (estimated CrCL)**	**Aztreonam-avibactam dose regimen**^**a**^			**Aztreonam**		**Avibactam**		**Joint PTA, %**^**b**^
		**LD**	**ELD**	**MD**	**Total *C***_**max**_**, mg/L**	**Total AUC**_**(0–24)**_**, mg·h/L**	**Total *C***_**max**_**, mg/L**	**Total AUC**_**(0–24)**_**, mg.h/L**	
Phase IIa Cohort 1	Normal (> 80 mL/min)	500/137 mg	1500/410 mg	1500/410 mg q6h	58.2	972	9.1	142	87.0
	Mild impairment (> 50 to ≤ 80 mL/min)	500/137 mg	1500/410 mg	1500/410 mg q6h	77.7	1363	12.0	197	96.2
Phase IIa Cohorts 2 and 3 and Phase III	Normal (> 80 mL/min)	500/167 mg	1500/500 mg	1500/500 mg q6h	58.9	982	11.3	175	91.1
	Mild impairment (> 50 to ≤ 80 mL/min)	500/167 mg	1500/500 mg	1500/500 mg q6h	78.0	1372	14.8	241	97.8
	Moderate impairment (> 30 to ≤ 50 mL/min)	500/167 mg	1500/500 mg	750/250 mg q6h	50.4	931	11.0	200	93.1^c^
	Severe impairment (> 15 to ≤ 30 mL/min)	675/225 mg	675/225 mg	675/225 mg q8h	86.2	864	15.3	257	91.6

*AUC*_*(0–24)*_ area under the plasma concentration versus time curve from 0 to 24 h, *C*_*max*_ maximum concentration, *CrCL* creatinine clearance, *ELD* extended loading dose, *LD* loading dose, *MD* maintenance dose, *MIC* minimum inhibitory concentration, *PTA*, probability of target attainment, *q6h* every 6 h, *q8h* every 8 h

^a^LD given as 30-min infusions; ELD and MD given as 3-h infusions. ELD is given immediately after the end of the LD infusion

^b^Probability of attaining the joint PK-PD target, defined as free plasma concentrations of aztreonam above 8 mg/L for 60% of each dosing interval (60% *f*T > 8 mg/L) and free concentrations of avibactam above 2.5 mg/L for 50% of each dosing interval (50% *f*T > 2.5 mg/L). Both targets needed to be achieved simultaneously to meet the joint target

^c^Exposures and joint PTA based on an aztreonam-avibactam ELD of 750/250 mg

Predictions based on Iteration 2 simulations found that joint PTA at MIC = 8 mg/L at steady-state was slightly lower (87%) for patients with normal renal function (estimated CrCL > 80 mL/min) receiving the Phase IIa Cohort 1 aztreonam-avibactam dose regimen compared with the Iteration 1 simulations (Table 4 and Fig. 1D). Patients with mild renal impairment (estimated CrCL > 50 to ≤ 80 mL/min) receiving the same doses had higher predicted aztreonam and avibactam exposures (steady-state AUC_(0–24)_ ratios of 1.40 for and 1.39, respectively) and joint PTA > 95% (Table 4). However, joint PTA > 90% for patients with normal renal function could be achieved by increasing the aztreonam-avibactam loading dose to 500/167 mg and extended loading and maintenance doses to 1500/500 mg (Table 4 and Fig. 1D). Accordingly, this increased dose regimen was assessed in patients with estimated CrCL > 50 mL/min in Phase IIa Cohort 2, and expanded into Phase IIa Cohort 3 following favorable safety and tolerability assessments for the first 10 patients enrolled in each of Cohorts 1 and 2 [10].

The exposure and PTA results for the aztreonam-avibactam loading and maintenance dose regimen selected for Phase IIa Cohorts 2 and 3 above (designated the ‘reference dose’) for patients with estimated CrCL > 50 mL/min were used as reference points for selecting dose regimens for patients with moderate and severe renal impairment (estimated CrCL > 30 to ≤ 50 mL/min and > 15 to ≤ 30 mL/min, respectively). Simulations for patients with moderate renal impairment evaluated aztreonam-avibactam maintenance doses of 45–70% of the daily 'reference dose' (maintaining a 3:1 ratio) administered by 3-h infusion q6h with either proportionate loading and extended loading doses, or with the higher ‘reference’ loading (500/167 mg [30-min infusion]) and extended loading doses (1500/500 mg [3-h infusion]). The aztreonam-avibactam maintenance dose for patients with moderate renal impairment that best matched exposures in patients with normal renal function was 750/250 mg q6h, giving steady-state AUC_(0–24)_ ratios of 0.95 for aztreonam and 1.14 for avibactam and joint PTA at MIC = 8 mg/L of 93.1% (Table 4). A proportionate loading dose of 250/83 mg and an extended loading dose of 750/250 mg gave joint PTA of approximately 74% in the first 6-h dosing period. Using the higher ‘reference’ loading (500/167 mg) and extended loading (1500/500 mg) doses for patients with moderate renal impairment resulted in joint PTA at MIC = 8 mg/L > 90% during the first 6-h dosing interval, albeit with transient increases in exposures relative to patients with normal renal function receiving the 'reference dose'. This regimen was therefore selected for patients with moderate renal impairment in the Phase IIa (Cohorts 2 and 3) and Phase III aztreonam-avibactam trials.

Simulations for patients with severe renal impairment evaluated aztreonam-avibactam maintenance doses of 30–40% of the daily ‘reference dose’ administered q6h, every 8 h (q8h) or every 12 h with proportionate loading and extended loading doses. A regimen comprising a loading dose of a 675/225 mg 30-min infusion, an extended loading dose of 675/225 mg 3-h infusion, and maintenance doses of 675/225 mg 3-h infusions q8h, giving steady-state AUC_(0–24)_ ratios of 0.88 for aztreonam and 1.47 for avibactam and joint PTA at MIC = 8 mg/L of 91.6% (Table 4), was selected for evaluation in the Phase III trials. A loading dose of 675/225 mg 30-min infusion followed immediately by an extended loading dose of 675/225 mg 3-h infusion for patients with severe renal impairment resulted in joint PTA at MIC = 8 mg/L of 90.6% during the first 8-h dosing interval, again with transient increases in exposures relative to the ‘reference dose’.

## Discussion

Dose regimen selection for the aztreonam-avibactam clinical program has been guided by approved dosing recommendations for aztreonam monotherapy, target pathogen MIC distributions for aztreonam and avibactam in combination, and an iterative process of population PK modeling and PTA simulations evaluating a range of dosing scenarios. A similar process of iterative population PK modeling was undertaken to support dose modification in order to ensure adequate dosing for patients with renal impairment in the ceftazidime-avibactam clinical program [24].

Aztreonam as a monotherapy is approved at doses up to 8 g/day, and is associated with elevations of plasma AST and ALT levels in some patients; such transaminase elevations are typically transient and asymptomatic, and normalize during treatment or following treatment discontinuation [39, 40]. In the Phase I aztreonam-avibactam dose-finding trial, various dose regimens were explored, including monitoring of transaminase effects, facilitated by a three-part study design with sequential cohorts of healthy subjects [9]. The analyses described here, which supported the selection and modification/optimization of aztreonam-avibactam dosages for clinical investigation, demonstrate the following: (1) aztreonam-avibactam extended (3-h) infusions and q6h dosing in most patients are optimal to achieve adequate exposures/joint PTA; (2) when accounting for patient variability, avibactam dose is the limiting factor for achieving the joint PTA target at MIC < 8 mg/L; and (3) a loading dose improves joint PTA in the first dosing interval. These analyses benefited from a large and robust population PK dataset for avibactam, including patients with cIAI and cUTI from the ceftazidime-avibactam clinical development program in Iteration 2 [21, 22]. In contrast, no large-scale population PK models for aztreonam in patients with complicated infections were available. Accordingly, for the simulations based on PK model Iteration 1, various assumptions were tested to make the aztreonam data more representative of infected patients, and for those based on PK model Iteration 2, scaling of *V*_*c*_ at steady state was assumed to be the same as that of avibactam in Phase III patients with cIAI (i.e., 27.5% higher). While a non-linear protein binding function for the aztreonam Iteration 2 population PK models provided the best fit to the data, concentration-independent binding of aztreonam to human plasma proteins has been reported [16] and confirmed in vitro over the range of clinically relevant concentrations (unpublished data). An alternative model (not presented) without non-linearity in the observed concentrations provided very similar CL and *V*_*c*_ parameter estimates. As such, more current modeling iterations use the simplified aztreonam PK model.

Exposure and joint PTA simulations were conducted using Iteration 1 PK models part way through the Phase I aztreonam-avibactam trial to support downward dose adjustments with the aim of achieving adequate PTA while reducing the potential for observed transaminase elevations. These analyses suggested that reduced aztreonam maintenance doses with extended (3-h) infusions administered q6h resulted in adequate joint PTA for the primary target (60% *f*T > 8 mg/L for aztreonam and 50% *f*T > 2.5 mg/L for avibactam); when renal impairment was considered, inclusion of a loading dose resulted in more simulated patients achieving the joint PK-PD target during the initial dosing interval. For the simulations based on the Iteration 2 PK models, scaling on *V*_*c*_ and variability of aztreonam CL and *V*_*c*_ used values from avibactam PK model Iteration 2, which included Phase III patients with cIAI or cUTI. The additional data allowed variability of avibactam in the patient population to be more robustly predicted; joint PTA at the target of MIC = 8 mg/L for the Phase IIa Cohort 1 dose regimen (loading 500/137 mg, maintenance 1500/410 mg q6h) in simulated patients with normal renal function was slightly lower than previously estimated based on the Iteration 1 simulations. The increased variability is the likely reason that Iteration 2 simulations models required an increase in avibactam dose to achieve joint PTA > 90%. Accordingly, increased loading (500/167 mg) and maintenance (1500/500 mg q6h) doses were selected for evaluation in Phase IIa Cohorts 2 and 3 and for the Phase III trials. Simulations for moderate and severe renal impairment using the Iteration 2 PK models also highlighted the importance of the loading dose for attaining joint PTA > 90% at MIC = 8 mg/L during the initial dosing interval (74% vs > 95% for a proportionate loading dose vs the 'reference' loading dose in patients with moderate renal impairment). Of note, aztreonam monotherapy doses are not adjusted for moderate renal impairment [28, 29]. Considering the differential renal clearance of the two drugs and the fixed 3:1 aztreonam to avibactam combination, the use of loading doses in patients with moderate or severe renal impairment to support achievement of adequate aztreonam exposures will inevitably produce transient increases in avibactam exposures beyond those predicted for patients with normal renal function. However, estimated avibactam exposures for the selected aztreonam-avibactam dose regimens for moderate or severe renal impairment are of similar magnitude to those in subjects with mild renal impairment and are thus considered unlikely to result in additional safety/tolerability risks.

Strengths of the current analyses include the large patient PK database for avibactam, and use of a simultaneous joint PTA target, which is more conservative than approaches used for some other antibiotic/inhibitor combinations [41, 42]. The use of Phase III cIAI avibactam data and assumptions from the ceftazidime-avibactam program to support the development of aztreonam-avibactam is justified by the lack of drug–drug interactions between ceftazidime and avibactam [43, 44] as well as the absence of interactions between aztreonam and avibactam demonstrated in the Phase I dose-finding trial [9]. The lack of patient PK data for aztreonam informing the model (particularly in respect of the critically ill patient population), and the use of a combination of (older) published PK data for subjects with renal impairment [32, 33] and (more recent) data from the Phase I trial (with different analytical methods for measuring aztreonam plasma concentrations) are nevertheless potential limitations of the current analyses. However, aztreonam PK data obtained from the literature also included some subjects with normal renal function [32, 33]; while not formally evaluated, the plasma concentrations were consistent with our Phase I observations in healthy subjects (data not shown). The absence of data for patients with severe hypoalbuminemia (nephrotic syndrome or other proteinuric diseases) limits the clinical applicability of these simulations. Moreover, models and PK simulations were based on calculation of free concentrations using a fixed protein-binding estimate for joint PTA calculations. Higher unbound drug fractions due to hypoalbuminemia could be expected to increase V and CL [45, 46].

Subsequent to the current analyses, a simultaneous population PK model for aztreonam and avibactam has been developed to support initial dose selection for pediatric clinical studies [47]. Future model iterations will include aztreonam plasma PK data from patients in the Phase IIa and Phase III aztreonam-avibactam clinical trials, and from a Phase I trial of aztreonam-avibactam in subjects with severe renal impairment (NCT04486625) and utilize simultaneous modeling to efficiently estimate correlations of PK variability between aztreonam and avibactam.

Given the severely limited available treatment options for infections caused by MBL-producing Gram-negative bacteria, there have been reports on the use of combinations of ceftazidime-avibactam plus aztreonam (frequently combined with other antibiotics) in severely ill hospitalized patients, with some evidence of effectiveness in single patient cases and small case series [48–50]. Based on these data, ceftazidime-avibactam plus aztreonam has been included in recent international treatment guidelines [51–53]; guidance from the Infectious Diseases Society of America (IDSA) includes a suggested dose regimen for ceftazidime-avibactam 2.5 g plus aztreonam 2 g by 3-h infusions q8h or q6h (q6h dosing is preferred if possible) [52–54]. While it is encouraging that positive outcomes have been reported for ceftazidime-avibactam plus aztreonam, the IDSA recommendation does not include a loading dose, and based on the current simulations, the proposed regimen would be unable to achieve sufficient avibactam exposures to ensure > 90% joint PTA for aztreonam-avibactam at MIC = 8 mg/L. As such there is a possibility of inadequate PK-PD target attainment (particularly at higher-pathogen MICs) for ceftazidime-avibactam plus aztreonam dosed according to the IDSA recommendation. Also, without loading doses, there will be on average a longer time to achieve targets in the critical early phase of treatment. In addition, the safety (including transaminase liability) of ceftazidime-avibactam plus aztreonam is unknown, although transaminase elevations have been reported to be more frequent with combinations including 8 g/day of aztreonam compared to those including 6 g/day [55]. Overall, the “off-label” use of combinations of ceftazidime-avibactam plus aztreonam might not provide adequate exposures for all patients across the range of target pathogen susceptibilities.

In summary, results from two influential iterations of population PK modeling and simulations for efficacy (based on joint PTA analyses) during and after completion of the aztreonam-avibactam Phase I dose-finding trial have supported selection and modification of aztreonam-avibactam dosage regimens (based on a 3:1 fixed ratio) for Phase IIa and Phase III clinical evaluation. The final selected aztreonam-avibactam dose regimen for Phase III patients with estimated CrCL > 50 mL/min is a 500/167 mg loading 30-min infusion followed immediately by 1500/500 mg maintenance 3-h infusions q6h. Adjustments for moderate renal impairment (estimated CrCL > 30 to ≤ 50 mL/min) and severe renal impairment (> 15 to ≤ 30 mL/min) account for the differential renal clearance of aztreonam and avibactam and share a consistent schedule of loading doses (30-min infusions) followed by extended loading doses and maintenance doses (3-h infusions) q6h or q8h, respectively.

### Supplementary Information

Below is the link to the electronic supplementary material.

Supplementary file1 (DOCX 85 KB)

## Data Availability

Upon request, and subject to certain criteria, conditions, and exceptions, see https://www.pfizer.com/science/clinical-trials/trial-data-and-results for more information. Pfizer will provide access to individual de-identified participant data from Pfizer-sponsored global interventional clinical studies conducted for medicines, vaccines and medical devices (1) for indications that have been approved in the US and/or EU or (2) in programs that have been terminated (i.e., development for all indications has been discontinued). Pfizer will also consider requests for the protocol, data dictionary, and statistical analysis plan. Data may be requested from Pfizer trials 24 months after study completion. The de-identified participant data will be made available to researchers whose proposals meet the research criteria and other conditions, and for which an exception does not apply, via a secure portal. To gain access, data requestors must enter into a data access agreement with Pfizer.
