# Supplementary material for: Dose selection for aztreonam-avibactam, including adjustments for renal impairment, for Phase IIa and Phase III evaluation
Source: Eur J Clin Pharmacol. 2024 Jan 22;80(4):529–43. doi: 10.1007/s00228-023-03609-x (PMC10937790; doi:10.1007/s00228-023-03609-x)
Supplement: Supplementary file 1 — Supplementary file1 (DOCX 85 KB) [file 228_2023_3609_MOESM1_ESM.docx]

# Supplementary appendix

Dose selection for aztreonam-avibactam, including adjustments for renal impairment, for phase IIa and phase III evaluation

Shampa Das,^1^* Todd Riccobene,^2^ Timothy J. Carrothers,^2**^ James G. Wright,^3^ Merran MacPherson,^3†^ Andrew Cristinacce,^3^ Lynn McFadyen,^4§^ Rujia Xie,^5^ Alison Luckey,^6‡^ Susan Raber^7^

^1^AstraZeneca, Alderley Park, Macclesfield, UK; ^2^AbbVie, Madison, NJ, USA; ^3^Wright Dose Ltd, Altrincham, Cheshire, UK; ^4^Pfizer, Sandwich, Kent, UK; ^5^Pfizer, Singapore; ^6^Pfizer, New York, NY, USA; ^7^Pfizer, La Jolla, CA, USA

*Present address: Department of Molecular and Clinical Pharmacology, University of Liverpool, Liverpool, UK
**Present address: Intra-Cellular Therapies, Inc., New York, NY, USA ^†^Present address: UCB, Braine-l’Alleude, Wallonia, Belgium
^§^Present address: Canterbury, Kent, UK ^‡^Present address: GARDP (Global Antibiotics Research & Development Partnership), Geneva, Switzerland

**Corresponding author:**Susan Raber, PharmD, MPH
Senior Director, Clinical Pharmacology
Global Product Development, Pfizer Inc. 10555 Science Center Dr., San Diego, CA, 92121, USA [susan.raber@pfizer.com](mailto:susan.raber@pfizer.com)

## Supplementary Table 1 Overview of aztreonam PK scenarios evaluated in simulations based on population PK model Iteration 1

|  | **Aztreonam PK parameter values (IIV%)** | |
| --- | --- | --- |
|  | **CL, L/h** | **V_c_, L** |
| **Case 1**  CL and V_c_ for healthy subjects from aztreonam PK model Iteration 1a (Supplementary Table 2) | 5.5 (8.8) | 7.93 (19) |
| **Case 2**  CL for patients with CF [1], IIV doubled from Case 1 | 6.1 (17.6) | 7.93 (19) |
| **Case 3**  CL for patients with CF, IIV from avibactam PK model Iteration 1 (Supplementary Table 3) | 6.1 (27.9) | 7.93 (19) |
| **Case 4**  CL 45% increase, V_c_ 166% increase and IIVs for avibactam from phase II cIAI patients from avibactam PK model Iteration 1 (Supplementary Table 3) | 8.0 (27.9) | 21.1 (32.1) |
| **Case 5**  CL 23% increase, V_c_ 108% increase and IIVs for ceftazidime from phase II cIAI patients [2] | 6.8 (37.4) | 16.5 (49.0) |

*cIAI* complicated intra-abdominal infection, *CL* clearance, *IIV* inter-individual variability, *V_c_* apparent volume of the central compartment

## Supplementary Table 2 Aztreonam population PK parameters: models adapted for Iteration 1a and 1b simulations

|  | **Estimate (RSE%)** | |
| --- | --- | --- |
| **Parameter** | **Iteration 1a (without renal impairment data)** | **Iteration 1b (with renal impairment data)** |
| θ_1_: CL*WT/70**0.75 (L/h) | 5.5 (2.0) | 5.3 (2.3) |
| θ_5_: CL (L/h) ((Baseline CrCL/120)** θ_5_) | 0.45 (22.3) | 0.43 (6.3) |
| θ_2_: V_c_ *WT/70 (L) | 7.93 (4.1) | 7.46 (4.0) |
| θ_3_: Q*WT/70**0.75 (L/h) | 5.77 (11.2) | 7.95 (8.4) |
| θ_4_: V_p_*WT/70 (L) | 5.71 (2.9) | 5.94 (3.1) |
| ηCL (%) | 8.8 (31) | 22 (19) |
| ηV_c_ (%) | 19 (27) | 27 (21) |
| ηQ (%) | 51 (63) | 48 (46) |
| Proportional error | 0.15 (12) | 0.14 (12) |

*CL* clearance, *IIV* inter-individual variability, *nCrCL*, body-surface area normalized creatinine clearance, *Q* inter-compartmental clearance, *PK* pharmacokinetics, *RSE* relative standard error, *V_c_* apparent volume of the central compartment; *V_p_* apparent volume of the peripheral compartment

## Supplementary Table 3 Aztreonam population PK parameters: model Iteration 2

| **Parameter** | **Estimate (RSE%)** | **IIV, % (RSE%)** |
| --- | --- | --- |
| θ_1_: CL*WT/70** 0.75 (L/h) | 5.21 (3.73) | 14.1 (12.5) |
| θ_2_: V_c_*WT/70 (L) | 6.83 (4.43) | 30.0 (25.4) |
| θ_3_: Q*WT/70** 0.75 (L/h) | 10.7 (12.2) | – |
| θ_4_: V_p_*WT/70 (L) | 5.76 (5.27) | 21.9 (20.5) |
| θ_5_: (CL + θ_5_*(CrCL–120)) | – | – |
| θ_5_: (CL + θ_5_*(CrCL–80)) | 0.0158 (21.3) |  |
| θ_6_: (B_max_) ^a^ | 3.24 (19.7) |  |
| θ_7_: (B_50_) ^a^ | 3.45 (19.8) |  |
| θ_8_: (IF (AGE>50) V_c_&V_p_ + θ_8_AGE) | NA |  |
| θ_8_: (IF (AGE>65) V_c_&V_p_*θ_8_AGE) | 0.779 (21.5) |  |
| θ_9_: (IF CrCL<80) CrCL= θ_5_* θ_9_*(CrCL–80) | 2.86 (26.3) |  |
| ω5: 5 (IOV CL) | 11.2 (20.6) |  |
| Proportional error ^b^ | 9.1 (25.3) |  |
| Additive error ^b^ | 0.271 (56.6) |  |

*B_max_* maximum binding capacity, *CL* clearance, *CrCL* creatinine clearance, *IIV* inter-individual variability, *IOV* inter-observation variability, *Q* inter-compartmental clearance, *PK* pharmacokinetics, *RSE* relative standard error, *V_c_* apparent volume of the central compartment, *V_p_* apparent volume of the peripheral compartment
^a^Ff=F where Ff = free concentration; Fb=F* θ_6_/(F+ θ_7_) =bound concentration; IPRED=F=Fb
^b^Reported as variance. B_50_, 50% of B_max_

## Supplementary Table 4 Avibactam population PK parameters: model adapted for Iteration 1 simulations

| **Parameter** | **Estimate (RSE%)** | **IIV, % (RSE%)** |
| --- | --- | --- |
| θ_1_: CL (L/h) | 12.2 (3.3) | 27.9 (27.0) |
| θ_2_: CL, estimate for dialysis patients (L/h) | 17.1 (9.8) | - |
| θ_3_: V_c_ (L) | 12.8 (2.3) | 32.1 (22.1) |
| θ_4_: Q (L/h) | 4.96 (5.8) | 19.1 (59.8) |
| θ_5_: V_p_ (L) | 6.99 (3.1) | 41.0 (21.2) |
| θ_6_: Ka | 0.459 (12.2) |  |
| θ_7_: F1 | 0.0656 (10.7) |  |
| θ_8_: CL, (nCrCL/80)**θ_8_, nCrCL <80 mL/min and not ESRD | 1.23 (6.4) |  |
| θ_9_: CL, Change in CL due to ESRD | 0.0447 (32.9) |  |
| θ_10_: CL*(WT/70)** θ_10_ | 0.206 (57.3) |  |
| θ_11_: V_c_*(WT/70)** θ_11_ | 0.516 (35.3) |  |
| θ_12_: Q*(WT/70)** θ_12_ | 1.33 (31.7) |  |
| θ_13_: V_p_*(WT/70)** θ_13_ | 1.23 (15.3) |  |
| θ_14_: AGE/35** θ_14_ on CL | –1.13 (47.3) |  |
| θ_15_: θ_15_**SEX on CL | 0.957 (3.6) |  |
| θ_16_: Concomitant ceftazidime-avibactam on CL | 0.963 (1.2) |  |
| θ_17_: θ_17_** phase II cIAI patient effect on CL | 1.45 (7.4) |  |
| θ_18_: θ_18_** phase II cIAI patient effect on V_c_ | 2.66 (11.2) |  |

*cIAI* complicated intra-abdominal infection, *CL* clearance, *cUTI* complicated urinary tract infection, *ESRD* end-stage renal disease, *F1* relative bioavailability for oral dosing, *IIV* inter-individual variability, *Ka* absorption rate, *nCrCL* body-surface area normalized creatinine clearance; *Q* inter-compartmental clearance, *PK* pharmacokinetics, *RSE* relative standard error, *V_c_* apparent volume of the central compartment, *V_p_* apparent volume of the peripheral compartment, *WT* body weight (kg)

Supplementary Table 5 Avibactam population PK parameters: model Iteration 2

| **Parameter** | **Estimate (RSE%)** | **IIV, %** |
| --- | --- | --- |
| θ_1_: CL (L/h) | 10.4 (1.8) | 55.9 |
| θ_2_: V_c_ (L) | 12.0 (9.8) | 100.4 |
| θ_3_: Q (L/h) | 4.99 (15.6) | 129.7 |
| θ_4_: V_p_ (L) | 6.66 (7.1) | 230.3 |
| θ_5_: ESRD effect on CL (CL*θ_5_) | 0.0671 (6.9) |  |
| θ_6_: CL, estimate for dialysis patients | 20.5 (7.9) |  |
| θ_7_: CL, (CrCL/80)**θ_7_, CrCL <80 mL/min | 1.1 (2.1) |  |
| θ_8_: CL, (1+θ_8_*(CrCL–80)), CrCL ≥80 mL/min | 0.00254 (4.1) |  |
| θ_9_: Population effect on V_c_ (cIAI, phase II), V_c_*(1+θ_9_) | 1.34 (25.8) |  |
| θ_10_: Population effect on CL (cIAI, phase II), CL*(1+θ_10_) | 0.305 (22.9) |  |
| θ_11_: Population effect on V_c_ (cUTI), V_c_*(1+θ_11_) | 0.394 (23.8) |  |
| θ_12_: Population effect on V_c_ (cIAI, phase III), V_c_*(1+θ_12_) | 0.275 (31.3) |  |
| θ_15_: V_c_* (WT/71.2)** θ_15_ | 0.998 (8.2) |  |
| θ_16_: APACHE effect on CL, CL*(1+θ_16_) | –0.2 (18.2) |  |
|  |  | **Shrinkage (%) or correlation^a^** |
| ηCL^2^ | 0.313 (2.3) | 7.5 |
| ηV_c_^2^ | 1.009 (7.3) | 32.8 |
| ηCL–ηV_c_ covariance^b^ | 0.091 (20.1) | r = 0.16 |
| ηV_p_^2^ | 1.683 (6.9) | 12.72 |
| ηV_p_–ηCL covariance^b^ | 0.625 (4.1) | r = 0.86 |
| ηV_p_–ηV_c_ covariance^b^ | –0.423 (16.9) | r = –0.32 |
| ηQ 2 | 5.302 (9.9) | 16.85 |
| ηQ–ηCL covariance^b^ | 0.962 (5.8) | r = 0.75 |
| ηQ–ηV_c_ covariance^b^ | –1.186 (12.9) | r = –0.51 |
| ηQ–ηV_p_ covariance^b^ | 2.911 (8.1) | r = 0.97 |
| Proportional error, phase I^b^ | 0.03 (0.3) | 3.5 |
| Additive error, phase I^b^ | 1957.67 (1.0) | 3.5 |
| Proportional error, phase II^b^ | 0.245 (6.0) | 1.8 |
| Proportional error, phase III^b^ | 0.143 (2.6) | 13.2 |

*APACHE* Acute Physiology and Chronic Health Evaluation, *cIAI* complicated intra-abdominal infection, *CL* clearance, *CrCL* creatinine clearance, *cUTI* complicated urinary tract infection, *CV* coefficient of variation, *ESRD* end-stage renal disease, *IIV* inter-individual variability, *Q* inter-compartmental clearance, *PK* pharmacokinetics, *RSE* relative standard error, *V_c_* apparent volume of the central compartment, *V_p_* apparent volume of the peripheral compartment, *WT* body weight (kg)
^a^Correlation coefficient (r) between random effects
^b^Reported as variance

# References

1. Vinks AA, van Rossem RN, Mathot RA, Heijerman HG, Mouton JW (2007) Pharmacokinetics of aztreonam in healthy subjects and patients with cystic fibrosis and evaluation of dose-exposure relationships using monte carlo simulation. Antimicrob Agents Chemother 51:3049-3055. <https://doi.org/10.1128/aac.01522-06>

2. Li J, Knebel W, Riggs M, Zhou D, Nichols WW, Das S (2012) Population pharmacokinetic modelling of ceftazidime (CAZ) and avibactam (AVI) in healthy volunteers and patients with complicated intra-abdominal infection (cIAI). A-634. Presented at: 52nd Interscience Conference of Antimicrobial Agents and Chemotherapy (ICAAC). San Francisco, CA, USA.
